# Ferulic acid ameliorates the quality of *in vitro*-aged bovine oocytes by suppressing oxidative stress and apoptosis

**DOI:** 10.18632/aging.205193

**Published:** 2023-11-08

**Authors:** Yi-Jing Yin, Yong-Hong Zhang, Yu Wang, Hao Jiang, Jia-Bao Zhang, Shuang Liang, Bao Yuan

**Affiliations:** 1 Department of Animals Sciences, College of Animal Sciences, Jilin University, Changchun, China

**Keywords:** ferulic acid, bovine, oocyte aging, oxidative stress, apoptosis

## Abstract

Ferulic acid (FA) is a well-known natural antioxidant that scavenges oxygen free radicals and alleviates oxidative stress. This study investigated the chemopreventive potential of FA against bovine oocyte quality decline during *in vitro* aging. The results showed that 5 μM FA supplementation decreased the abnormality rate of *in vitro*-aged bovine oocytes. In addition, FA supplementation effectively improved antioxidant capacity by removing excessive ROS and maintaining intracellular GSH levels and antioxidant enzyme activity. The mitochondrial activity, mitochondrial membrane potential and intracellular ATP levels in aged bovine oocytes were obviously enhanced by FA supplementation. Furthermore, FA supplementation reduced *in vitro* aging-induced DNA damage and maintained DNA stability in bovine oocytes. Moreover, sperm binding assay showed the number of sperm that bound to the zona pellucida on aged bovine oocytes was significantly higher in the FA supplemented group than in the Aged group. Therefore, FA is beneficial for maintaining *in vitro*-aged bovine oocyte quality and could become a potential antioxidant for preventing bovine oocyte *in vitro* aging during *in vitro* maturation.

## INTRODUCTION

In the process of oocyte maturation *in vivo* or *in vitro*, oocytes in the metaphase of the second meiosis (MII) phase undergo time-related quality degradation if they are not fertilized in time [1]. *In vivo*, the inability to accurately predict the optimal fertilization time causes a delay in fertilization. Consequently, the oocytes can be retained in the oviduct after ovulation, which may cause oocyte aging [2]. *In vitro*, oocytes need to be cultured to maturation before micromanipulation and *in vitro* fertilization. Nevertheless, variations in individual oocytes result in distinct maturation durations, and extending the culture time is inevitable. This, in turn, contributes to oocyte aging [3].

Oocyte aging substantially diminishes fertilization rates and subsequent embryonic development potential [1], whilst also increasing the risk of miscarriage and fetal malformation [4]. Oocyte aging adversely affects oocyte quality mainly in terms of morphology and organelles as well as biochemical and molecular perspectives [5]. In terms of morphology and organelles, aging oocytes exhibit perivitelline space (PVS) increases, first polar body degradation [6], zona pellucida (ZP) hardening [7], chromosome disorder [8] and spindle morphological abnormalities [9]. From the biochemical and molecular perspectives, aging is often accompanied by excessive intracellular ROS accumulation [10], GSH levels reduction [11] and Ca^2+^ oscillation signal disorder [12]. There is much evidence revealing the close relationship between aging and ROS [13–15]. Excessive accumulation of ROS causes oxidative damage to DNA, proteins, and lipids, and the accumulation of oxidative damage is a common feature of aging [16–18]. As a matter of fact, aging-induced oxidative damage typically results in the malfunction or deactivation of multiple enzymes, which in turn causes DNA damage. The effects of DNA damage are varied. The blockage of gene transcription and DNA replication can result in various adverse effects, including cellular dysfunction or apoptosis [19]. With the gradual deepening of research on aging, especially oocyte aging, it has been found that supplementation with antioxidants during *in vitro* aging period can effectively delay oocyte aging, such as melatonin and coenzyme Q10 [20–23].

Ferulic acid (FA; 4-hydroxy-3-methoxycinnamic acid) is a natural antioxidant that is widely present in the cell walls of monocotyledonous plants [24]. It mainly prevents the occurrence of oxidative stress by scavenging excessive intracellular ROS [25]. In addition, FA has been shown to have antiaging effects [26]. Since aging is often accompanied by oxidative stress [10], we hypothesized that FA can delay oocyte aging and improve oocyte quality by resisting oxidative stress.

Here, we investigated the effect of FA on the abnormality rate of aging in bovine oocytes and evaluated the antioxidant capacity, mitochondrial activity and membrane potential (MMP), ATP levels, apoptosis and sperm binding capacity of *in vitro*-aged bovine oocytes. Our results will help to clarify the molecular mechanism of oocyte quality control and provide some data support and reference value for delaying oocyte aging and improving animal reproduction.

## RESULTS

### FA palliates aging-induced oocyte morphological anomalies

**Figure 1:**
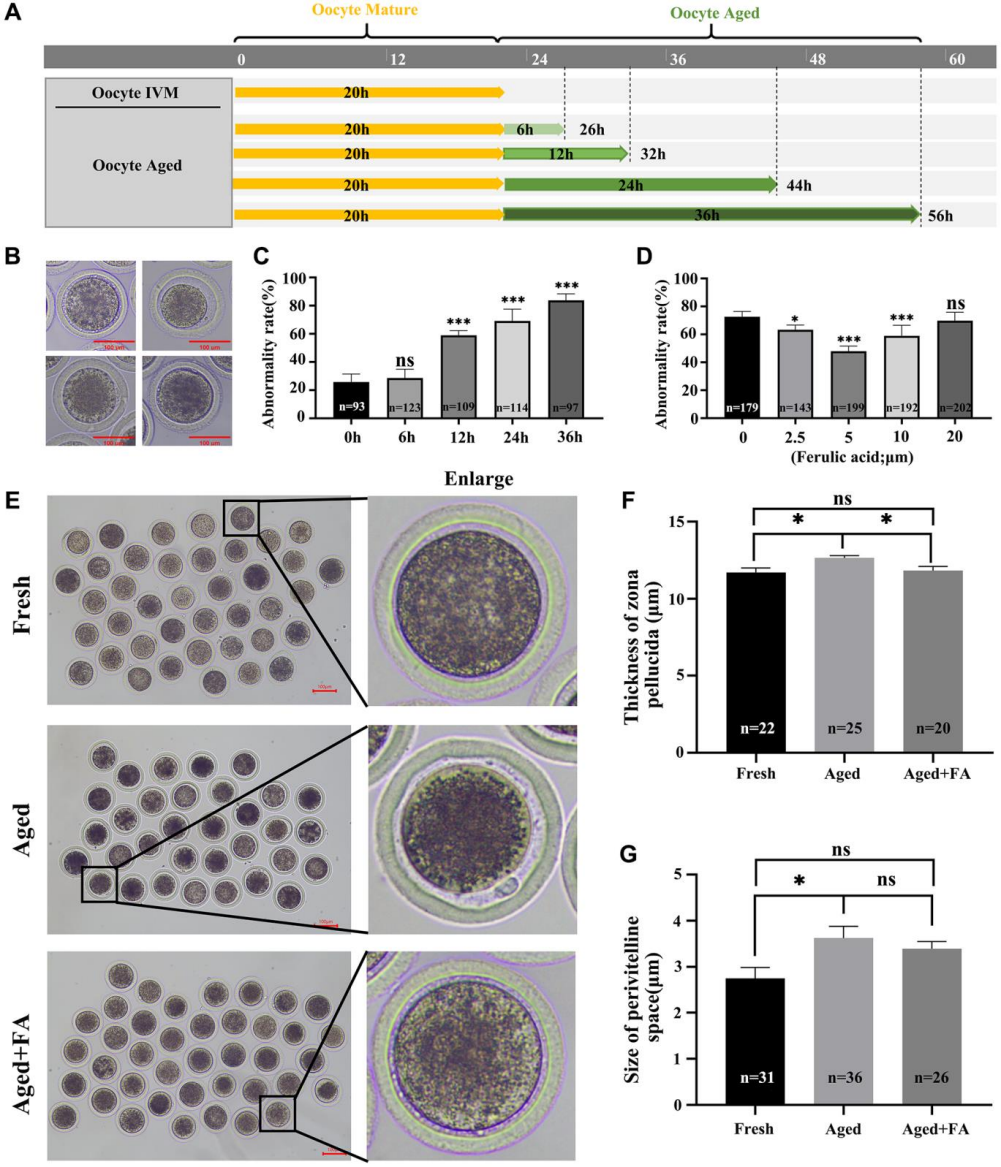
**FA palliates aging-induced oocyte morphological anomalies.** (**A**) Timeline diagram of *in vitro*-aged bovine oocytes. (**B**) Representative images of oocyte morphological anomalies (a very granular PVS, large PVS, first polar body degradation, and nonuniform cytoplasm). (**C**) The abnormality rates of *in vitro* aging for 6 h, 12 h, 24 h and 36 h groups. *R* = 4. (**D**) The abnormality rates of oocytes *in vitro* aged 12 h treated with different concentrations of FA (0, 2.5, 5, 10, or 20 μM). *R* = 7. (**E**) Representative images of PVS morphology in the Fresh, Aged, and Aged + FA groups. Scale bars: 100 μm. (**F**, **G**) Thickness of ZP and size of PVS of Fresh, Aged, Aged + FA groups. ^*^P < 0.05, ^**^P < 0.01, and ^***^P < 0.001 indicate significant differences.

After 20–22 h of *in vitro* culture, the oocytes were matured and categorized as the Fresh group. The culture time was prolonged to achieve *in vitro* aging for 6 h, 12 h, 24 h and 36 h. These oocytes were used and categorized as the Aged group (Figure 1A). Here, we detected the abnormality rate of oocytes. In this study, oocytes with a very granular PVS, large PVS, first polar body degradation, or nonuniform cytoplasm were considered as abnormal oocytes based on the observed oocyte morphology (Figure 1B). As shown in Figure 1C, there was a positive correlation between the abnormality rate of oocytes and the time of *in vitro* culture. Compared with that of the Fresh group (25.78 ± 2.83%, *n* = 93), the abnormality rates of oocytes aged for 12 h and above were significantly increased (*in vitro* aging for 12 h: 59.03 ± 1.46%, *n* = 109, *P* < 0.001; 24 h: 69.13 ± 3.77%, *n* = 114, *P* < 0.001 and 36 h: 83.78 ± 2.30%, *n* = 97, *P* < 0.001). In order to ensure the proper conduct of subsequent experiments, *in vitro* aging for 12 h was selected for further studies.

To explore the effect of FA on the abnormality rate of oocytes during *in vitro* aging, different concentrations of FA (0, 2.5, 5, 10 and 20 μM) were supplemented during *in vitro* aging process. As shown in Figure 1D, compared with the control group (72.74 ± 1.41%, *n* = 179), the 2.5, 5 and 10 μM FA treatment groups had significantly lower abnormality rates of aging oocytes (2.5 μM: 63.36 ± 1.38%, *n* = 143, *P* < 0.05; 5 μM: 47.93 ± 1.30%, *n* = 199, *P* < 0.001; 10 μM: 59.08 ± 2.64%, *n* = 192, *P* < 0.001). Among them, the abnormality rate of aging oocytes in the 5 μM treatment group was the lowest. Therefore, a concentration of 5 μM was selected for subsequent studies.

The ZP thickness and PVS size are important indicators for evaluating whether oocytes are abnormal and for evaluating subsequent embryonic development. Here, we used existing evaluation methods to calculate the above two indices (Supplementary Figure 1) [27]. The results were shown in Figure 1E–1G. Compared with those in the Aged group (thickness: 12.64 ± 0.81 μm, *n* = 25; size: 3.83 ± 1.64 μm, *n* = 36), the thickness of the ZP (11.82 ± 1.22 μm, *n* = 20, *P* < 0.05) was significantly reduced, the size of the PVS (3.65 ± 1.00 μm, *n* = 26) showed no obvious change in the FA treatment group and higher than those in the Fresh group (thickness: 11.70 ± 1.36 μm, *n* = 22; size: 2.42 ± 1.34 μm, *n* = 31). The above results showed that FA could effectively alleviate aging-induced bovine oocytes morphological abnormality.

### FA relieves aging-induced oocyte oxidation resistance

**Figure 2:**
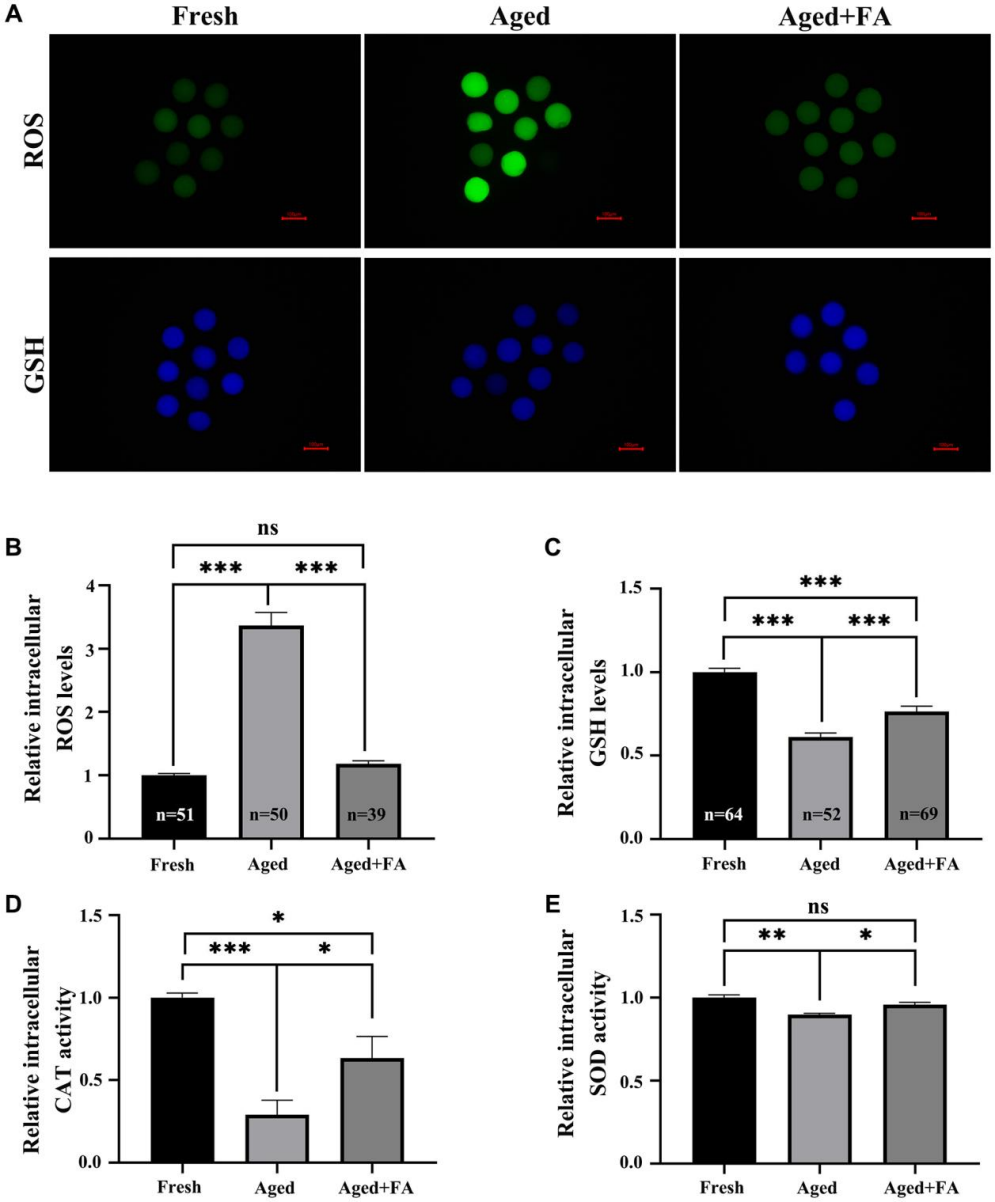
**FA relieves aging-induced oocyte oxidation resistance.** (**A**) Oocytes were stained with DCFH and CMF2HC to detect the intracellular ROS and GSH levels. Scale bar: 100 μm. *R* = 3. (**B**, **C**) Relative intracellular levels of ROS and GSH in bovine oocytes of the three groups (Fresh, Aged and Aged + FA group). (**D**, **E**) Relative intracellular activity of CAT and SOD in bovine oocytes from the three groups (Fresh, Aged, and Aged + FA). *R* = 4. ^*^P < 0.05, ^**^P < 0.01, and ^***^P < 0.001 indicate significant differences.

To explore the effect of FA on the antioxidant capacity of *in vitro*-aged oocytes, DCFH and CMF2HC were used to detect intracellular ROS and GSH levels, respectively. As shown in Figure 2A–2C, compared with those in Fresh group (ROS: 1.00 ± 0.03, *n* = 51; GSH: 1.00 ± 0.02, *n* = 64), the ROS levels in Aged group were significantly increased (3.36 ± 0.21, *n* = 50, *P* < 0.001), and the GSH levels were significantly decreased (0.61 ± 0.02, *n* = 52, *P* < 0.001). After FA supplementation, the levels of ROS in aged oocytes decreased significantly (1.18 ± 0.05, *n* = 39, *P* < 0.001), and the levels of GSH increased significantly (0.76 ± 0.03, *n* = 69, *P* < 0.001). To further explore the effect of FA on the antioxidant capacity of aging oocytes, we detected intracellular CAT and SOD activity. As shown in Figure 2D, 2E, the activity of CAT (0.29 ± 0.88, *P* < 0.001) and SOD (0.90 ± 0.01, *P* < 0.01) in the oocytes of the Aged group was significantly lower than that in the oocytes of the Fresh group (CAT: 1.00 ± 0.03; SOD: 1.00 ± 0.02), and the activity of the above two enzymes was significantly increased after FA supplementation (CAT:0.63 ± 0.13, *P* < 0.05; SOD:0.96 ± 0.02, *P* < 0.05). The above results indicated that FA could enhance the antioxidant capacity of aging oocytes.

### FA alleviates aging-induced oocyte mitochondrial dysfunction

**Figure 3:**
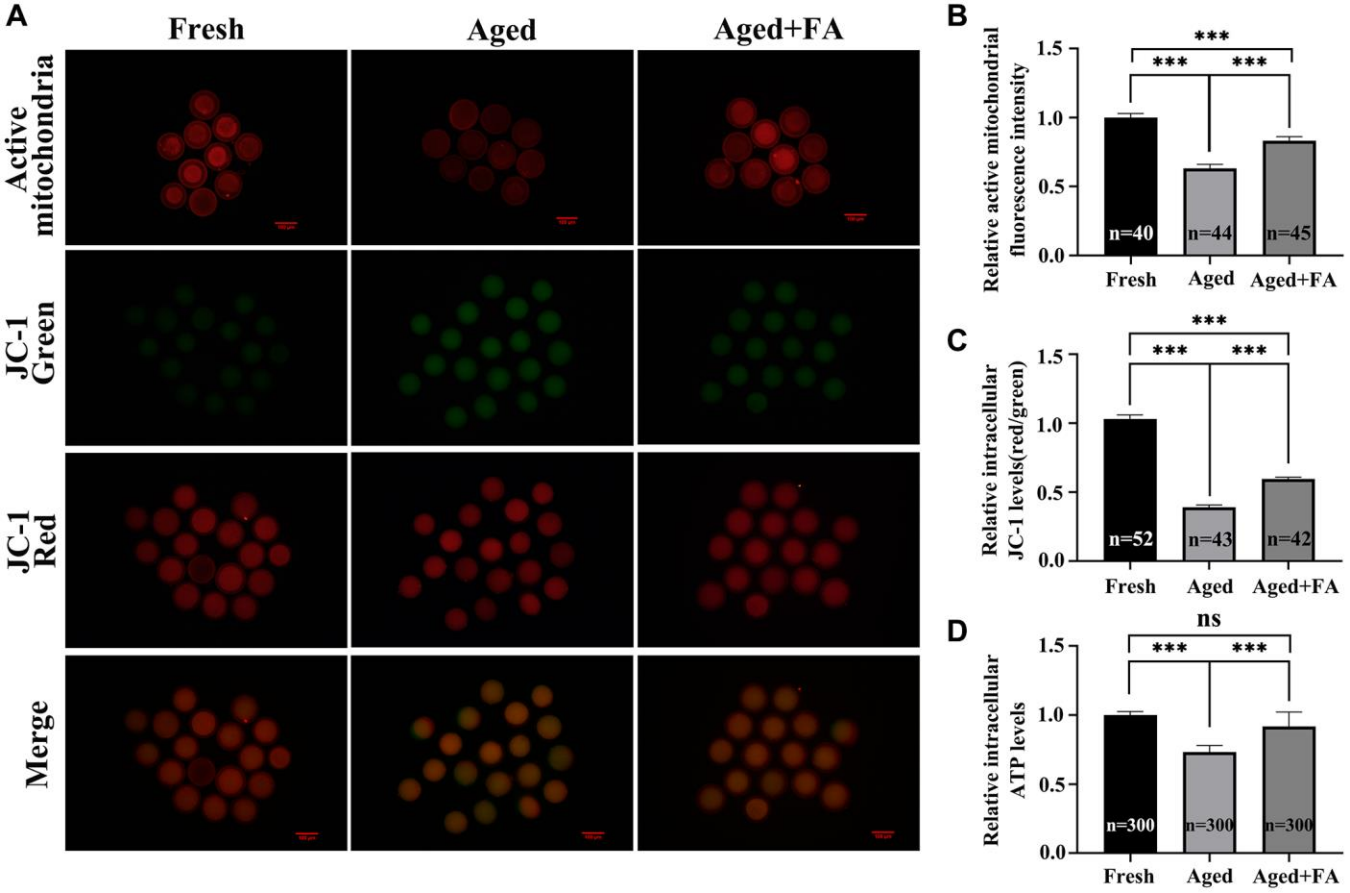
**FA alleviates aging-induced oocyte mitochondrial dysfunction.** (**A**) Oocytes were stained with MitoTracker Red CMXRos and JC-1 dyes to detect intracellular mitochondrial activity and MMP levels. Scale bar: 100 μm. *R* = 3. (**B**, **C**) Relative intracellular levels of active mitochondria and MMP in bovine oocytes from the three groups (Fresh, Aged, and Aged + FA group). (**D**) Relative intracellular ATP levels in bovine oocytes from the three groups (Fresh, Aged, and Aged + FA group). *R* = 6. ^*^P < 0.05, ^**^P < 0.01, and ^***^P < 0.001 indicate significant differences.

We used MitoTracker Red CMXRos and JC-1 dyes to stain oocytes to detect mitochondrial activity and MMP levels in oocytes, respectively. It is known to all that JC-1 monomers accumulate in mitochondria and form red fluorescent “J-aggregates” at a high MMP. At mitochondrial transmembrane potentials depolarised at low MMP, JC-1 exists as a green fluorescent monomer (Figure 3A). As shown in Figure 3A–3C, compared with those in the Fresh group (mitochondrial activity: 1.00 ± 0.03, *n* = 40; MMP: 1.03 ± 0.03, *n* = 52), the mitochondrial activity and MMP levels of oocytes in the aged group were significantly decreased (mitochondrial activity: 0.63 ± 0.03, *n* = 44, *P* < 0.001; MMP: 0.39 ± 0.02, *n* = 43, *P* < 0.001). After FA supplementation, the mitochondrial activity and MMP levels of aging oocytes were significantly increased (mitochondrial activity: 0.83 ± 0.03, *n* = 45, *P* < 0.001; MMP: 0.60 ± 0.01, *n* = 42, *P* < 0.001).

A decrease in MMP is often accompanied by changes in mitochondrial function, so we analyzed the intracellular ATP levels. As shown in Figure 3D, compared with those in the Fresh group (1.00 ± 0.01, *n* = 300), the ATP levels of oocytes in the Aged group were significantly decreased (0.73 ± 0.02, *n* = 300, *P* < 0.001). After FA supplementation, the ATP levels of aging oocytes were significantly increased (0.92 ± 0.04, *n* = 300, *P* < 0.001).

### FA mitigates aging-induced cellular senescence and DNA damage

**Figure 4:**
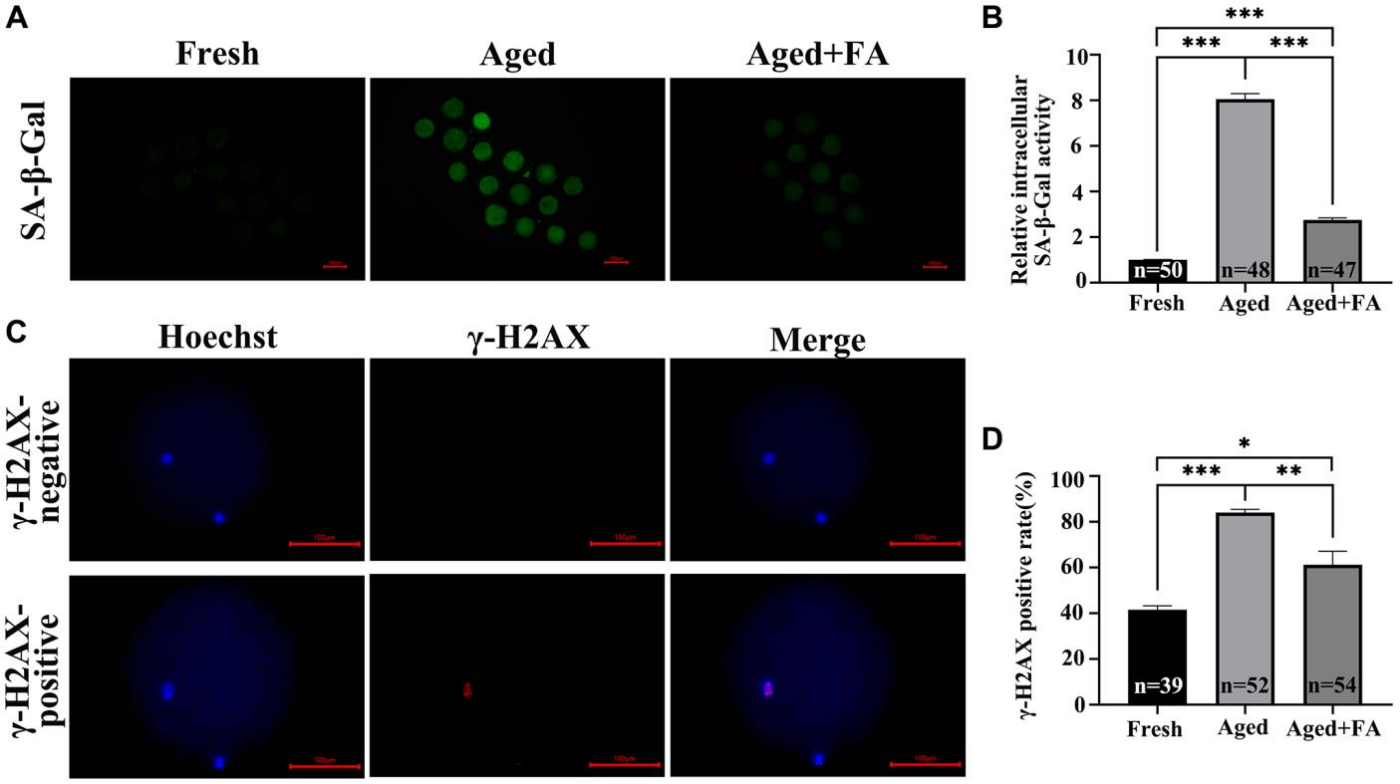
**FA mitigates aging-induced cellular senescence and DNA damage.** (**A**) Representative fluorescence images of intracellular SA-β-Gal activity in the three groups (Fresh, Aged, and Aged + FA). Scale bar: 100 μm. *R* = 3. (**B**) Relative intracellular levels of SA-β-Gal in bovine oocytes from the three groups (Fresh, Aged, and Aged + FA group). (**C**) Representative fluorescence images of positive and negative γ-H2AX staining. Scale bar: 100 μm. *R* = 3. (**D**) The γ-H2AX positivity rate in bovine oocytes from the three groups (Fresh, Aged, and Aged + FA group). ^*^P < 0.05, ^**^P < 0.01, and ^***^P < 0.001 indicate significant differences.

To explore the effect of FA on the cellular senescence of aging oocytes, we detected SA-β-Gal activity in oocytes. As shown in Figure 4A, 4B, the SA-β-Gal activity of oocytes in the Aged group was significantly higher than that of fresh oocytes (Aged group: 8.05 ± 0.26, *n* = 48; Fresh group: 1.00 ± 0.02, *n* = 50, *P* < 0.001). After FA supplementation, the activity of SA-β-Gal in aging oocytes was significantly decreased (2.76 ± 0.09, *n* = 47, *P* < 0.001).

As aging occurs, DNA damage accumulates. This will reduce the stability of DNA double strands, which will lead to the decrease of oocyte quality. Therefore, we examined the expression of the DNA double-strand damage repair marker γ-H2AX among the three groups (Figure 4C). As shown in Figure 4D, compared with that in the Fresh group (41.66 ± 1.61%, *n* = 39), the proportion of γ-H2AX-positive oocytes in the Aged group was significantly higher (84.16 ± 1.40%, *n* = 52, *P* < 0.001), while the positive proportion decreased to 61.22 ± 5.98% after FA supplementation (*n* = 54, *P* < 0.01).

The above results indicated that FA reduced *in vitro* aging-induced DNA damage and breakage and maintained DNA stability.

### FA inhibits aging-induced oocyte apoptosis

**Figure 5:**
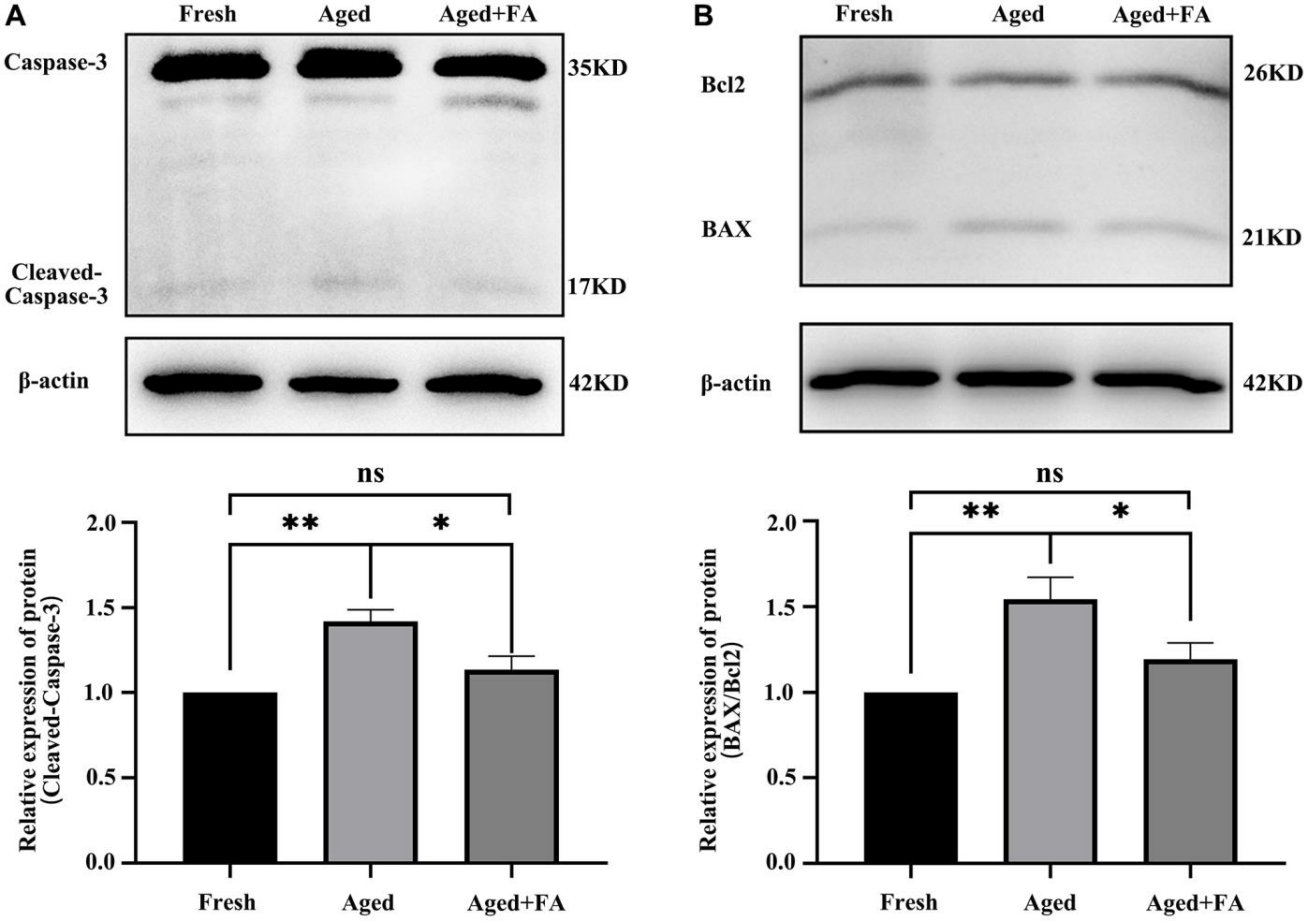
**FA inhibits aging-induced oocyte apoptosis.** (**A**, **B**) Representative Western blot images and relative expression levels of cleaved caspase-3 and BAX/Bcl2 in the three groups (Fresh, Aged, and Aged + FA group). *R* = 4. ^*^P < 0.05, ^**^P < 0.01, and ^***^P < 0.001 indicate significant differences.

Persistent DNA damage is a trigger for apoptosis. To evaluate whether FA inhibited the apoptosis of aging oocytes, we detected the protein expression levels of cleaved caspase-3, Bax and Bcl2 (Figure 5). Western blot analysis showed that the expression levels of cleaved caspase-3 (1.42 ± 0.07, *P* < 0.01) and BAX/Bcl2 (1.54 ± 0.13, *P* < 0.01) in aged oocytes were significantly higher than those in fresh oocytes, while the expression levels of cleaved caspase-3 (1.14 ± 0.08, *P* < 0.05) and BAX/Bcl2 (1.19 ± 0.10, *P* < 0.05) in aged oocytes were significantly decreased after FA supplementation. The above results indicated that FA supplementation could inhibit the apoptosis of aging oocytes.

### FA improves the sperm-binding ability of aged oocytes

**Figure 6:**
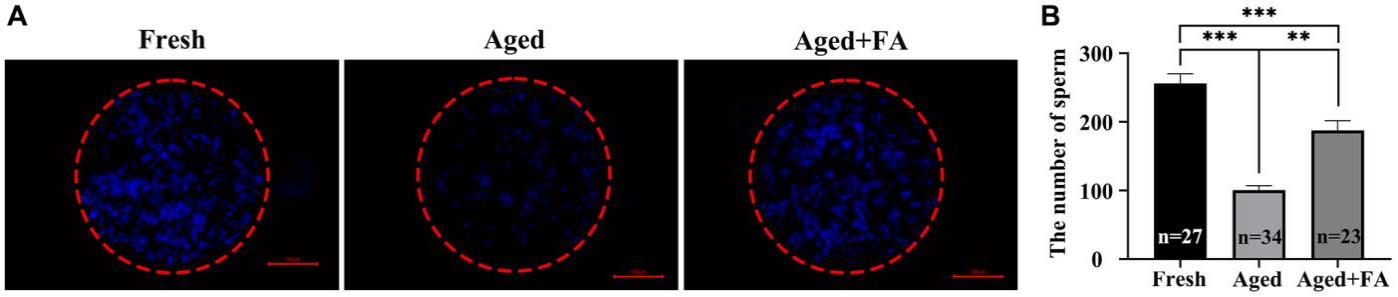
**FA improves the sperm binding ability of aged oocytes.** (**A**) Representative fluorescence images of sperm binding to the surface of the zona pellucida surrounding oocytes stained with Hoechst 33342 from the three groups (Fresh, Aged, and Aged + FA group). Scale bar: 100 μm. *R* = 3. (**B**) Number of sperm binding to the surface of the zona pellucida surrounding oocytes from the three groups (Fresh, Aged, and Aged + FA group). ^*^P < 0.05, ^**^P < 0.01, and ^***^P < 0.001 indicate significant differences.

To explore the effect of FA on the fertilization ability of aging oocytes, we detected the number of sperm bound to oocytes by sperm-oocyte binding analysis (Figure 6). The results showed that compared with that of the Fresh group (256.44 ± 14, *n* =27), the number of sperm bound to the ZP of oocytes in the aged group was significantly reduced (100.76 ± 6.26, *n* = 34, *P* < 0.001). After FA supplementation, the number of ZP-bound sperm in aged oocytes increased significantly (187.70 ± 14.26, *n* = 23, *P* < 0.01). The above results showed that FA supplementation could improve the fertilization ability of bovine oocytes.

## DISCUSSION

IVM is a significant part of assisted reproductive technology (ART), and oocyte quality exerts an important effect on IVM efficiency [28]. When the *in vitro* culture time is prolonged, the oocyte quality decreases in a time-dependent manner [29]. Many studies have shown that the optimal culture time of bovine oocytes are 20–22 h [30–32]. Here, we extended the culture time to set up an *in vitro* aging model, which demonstrated the positive effect of FA on *in vitro*-aged bovine oocytes.

Extensive results have shown that morphological abnormalities occur during IVM, especially after extended IVM period. These include a rise in the size of the PVS, an increase in debris within the PVS [6], fragmentation of the first polar body [33] and thickening of the ZP [34]. In this study, we observed the morphology of aged bovine oocytes at different culture time and treatment concentrations. In line with previous studies, our results suggest that the abnormality rate of oocytes increases in a time-dependent manner [29]. Moreover, FA supplementation has the potential to ameliorate the morphological abnormalities of aged oocytes.

The balance between ROS and GSH is essential for maintaining normal cell function [35]. ROS are widely involved in biological processes such as follicular development, meiosis, ovulation and embryonic development [36]. However, prolonged *in vitro* culture time can lead to excessive accumulation of ROS in unfertilized oocytes [37], resulting in oxidative stress that compromises oocyte quality [38, 39]. As a nonenzymatic antioxidant, GSH is responsible for cleaning up the excessive ROS to maintain cellular redox balance to alleviate intracellular oxidative stress [40] and improve the antioxidant capacity of aged oocytes [3, 41, 42]. In this study, we found that FA supplementation alleviated the increase in intracellular ROS and the decrease in GSH caused by oocyte aging. In addition, studies have shown that, as important members of antioxidative defense, the activity of CAT and SOD will decrease with the aging process [41, 43]. Meanwhile, FA can reduce intracellular ROS levels by increasing the activity of the above two antioxidant enzymes, thereby alleviating oxidative stress in oocytes [44, 45]. As expected, the activity of CAT and SOD in aged oocytes was increased after FA supplementation. The results of the experiment provide clear support for the statement that FA improves *in vitro*-aged bovine oocyte quality by resisting oxidative stress.

Mitochondria are the power sources of oocyte. They enable diverse physiological activities of cells by synthesizing ATP and have a fundamental role in oocyte maturation, fertilization and subsequent embryonic development [46–48]. There is mounting evidence indicating that mitochondrial dysfunction, as a cause or consequence of oxidative stress, is intrinsically linked to the process of aging [49]. Studies have shown that significant reductions in mitochondrial activity, MMP level and ATP production in aging oocytes can severely affect oocyte quality, which in turn leads to a decrease in oocyte development potential [50–52]. Our results showed that FA alleviated the decrease in MMP and partially restored mitochondrial function in aged oocytes. These results provide evidence that exogenous antioxidant supplementation can improve mitochondrial function in aged oocytes and thus promote oocyte developmental potential [42, 53, 54].

Aging-induced oocyte mitochondrial dysfunction can easily cause DNA damage in cells [55]. γH2AX, a biomarker of DNA double-strand breaks, recruits DNA repair proteins at the end of broken chromosomes to repair DNA damage [56, 57]. Previous studies have shown that aging can lead to a significant increase in the amount of γH2AX in cells [58, 59], which is consistent with our results. After FA supplementation, we observed a significant decrease in the γH2AX positive proportion. This suggests that FA has the potential to alleviate DNA double-strand breaks induced by aging and maintain the stability of DNA double-strands.

One of the prevalent cell responses to DNA damage is programmed cell death, or apoptosis [60, 61]. Caspase-3 is a crucial zymogen during cellular apoptosis, and is activated by cleavage during this process [62]. The antiapoptotic protein Bcl2 and the proapoptotic protein BAX induce apoptosis by permeabilizing the mitochondrial outer membrane (OMM) and then initiating the caspase cascade [63]. Our study found that the levels of cleaved caspase-3 and BAX/Bcl2 in aged oocytes were significantly increased, which was consistent with the findings of previous studies [64, 65]. After FA supplementation, the levels of the above apoptosis-related proteins were significantly reduced. These results clearly support our hypothesis that FA can protect oocytes against *in vitro* aging-induced apoptosis.

Sperm binding ability is one of the indicators used to evaluate the quality of oocytes. Since the complex process of fertilization begins with the binding of sperm to the ZP [66], we evaluated the sperm binding ability of oocytes via a sperm-oocyte binding assay. Here, we found that FA can increase the number of sperm bound to aging oocytes. Studies have shown that oocyte aging is usually accompanied by changes in the ZP [4]. It has been confirmed in mouse oocytes that postovulatory aging can lead to abnormal distribution of cortical granules and ovastacin in oocytes, resulting in premature cleavage of ZP2 before fertilization, thus hindering the normal binding of sperm to oocytes [67]. Therefore, we speculated that FA might improve the binding ability of sperm by alleviating premature exocytosis of aged oocytes.

**Figure 7:**
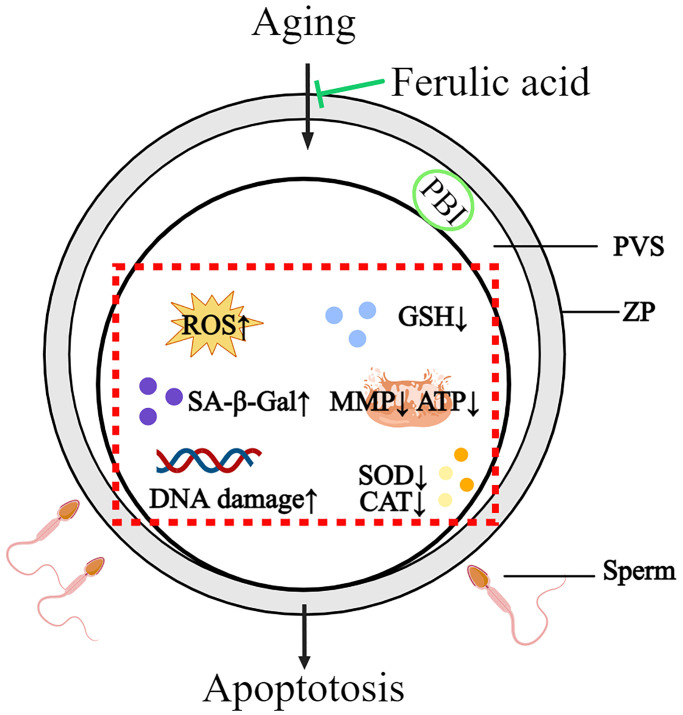
**Schematic diagram of the protective action of FA on *in vitro*-aged bovine oocytes.** After FA supplementation, intracellular ROS, SA-β-Gal, and DNA damage were decreased, while intracellular GSH, activity of CAT and SOD as well as mitochondria activity and function (MMP, ATP production) were increased in aged oocyte. These may help oocyte delay aging process and improve oocyte quality.

In summary, this study revealed that bovine oocytes *in vitro* aged may lead to a series of molecular events in oocytes, including oocyte morphological abnormalities, oxidative damage, mitochondrial dysfunction, increasing apoptosis and decreasing sperm-oocyte binding ability. FA supplementation could effectively improve the quality of *in vitro*-aged bovine oocytes by improving the antioxidant capacity, ameliorating mitochondrial function and inhibiting apoptosis. The above results indicate that FA may be useful for delaying oocyte aging in other mammals and provide new ideas for improving oocyte quality (Figure 7).

## MATERIALS AND METHODS

### Chemicals and reagents

Unless otherwise specified, all chemicals and reagents were purchased from Sigma-Aldrich (St. Louis, MO, USA). FA (CymitQuimica, Spain, CAS: 537-98-4) was diluted with DMSO to working concentrations of 2.5 μM, 5 μM, 10 μM and 20 μM. The control group was treated with the same concentration of DMSO.

### *In vitro* maturation and aging of bovine oocytes

Bovine ovaries were collected from the local slaughterhouse and transported to the laboratory within 2 hours at 37.5°C in normal saline supplemented with 1% penicillin G (75 mg/L) and streptomycin sulfate (50 mg/L). Cumulus oocyte complexes (COCs) were extracted from follicles with a diameter of 2–8 mm using 10 mL disposable syringes with an 18-gauge needle. Under a stereomicroscope (Zeiss, Stemi 305), oocytes wrapped in three or more intact cumulus layers were selected, washed three times in HEPES, placed in *in vitro* maturation (IVM) medium (tissue culture medium 199 supplemented with 100 mM Na pyruvate, 10 ng/mL EGF, 10% fetal bovine serum, 10 IU/mL follicle-stimulating hormone, 10 IU/mL luteinizing hormone and 2 μg/mL β-Estradiol), covered with mineral oil (Sage, ART-4008-5P) and placed in an environment under 38.5°C and 5% CO2 until maturation. For subsequent experiments, all experiments were performed on the basis of using 0.2% hyaluronidase to remove the cumulus cells of naked oocytes.

### *In vitro* aging treatment

Oocytes were cultured to maturity (*in vitro* culture for 20 h), and the culture time was extended to 26, 32, 44 and 56 h to observe the abnormality rates. Subsequent experiments were carried out on naked oocytes.

### Intracellular ROS and GSH level assay

To determine intracellular ROS and GSH levels, oocytes were treated with 2.5 μg/L 2′,7′-dichlorodihydrofluorescein diacetate (DCFH; S0033, Beyotime, China) or 10 μM 4-chloromethyl-6,8-difluoro-7-hydroxycoumarin (CMF2HC; C12881, Invitrogen, USA) and incubated in PBS-PVA medium at 37°C for 30 min. After washing the oocytes in PBS-PVA three times, the fluorescence intensity of each group of oocytes was captured by using a fluorescence microscope (Nikon, S22-LGB) and photographed. The fluorescence intensity was analyzed using ImageJ software (NIH, Stapleton, NY, USA).

### Superoxide dismutase and catalase activity assay

The superoxide dismutase (SOD) activity and catalase (CAT) activity were measured using a WST-8 total superoxide dismutase detection kit (Beyotime, S101S) and a catalase detection kit (BC0205, Solarbio, China). After preparing the standard reaction solution and curve, 40 oocytes were dissolved in the relevant lysis buffer and incubated with the reaction buffer for 30 min. Finally, the absorbance values were measured by a microplate reader (SpectraMax i3× Multi-Mode Detection Platform, Molecular devices, China), and the activity of SOD and CAT was calculated based on the absorbance values and standard curves.

### Mitochondrial activity assay

To assess mitochondrial activity, oocytes were incubated in the IVM medium accompanying MitoTracker Red CMXRos (Invitrogen, M7512) at 37°C for 30 min. After washing three times in PBS-PVA, the fluorescence intensity of each group of oocytes was captured by fluorescence microscope and photographed, and the fluorescence intensity was analyzed by ImageJ software.

### MMP assay

To determine the level of MMP, oocytes were incubated in PBS-PVA containing 2 μM 5,5′,6,6′-tetrachloro-1,1′,3,3′-tetraethylbenzimidazolylcarbocyanine iodide dye (JC-1; Beyotime, C2006) at 37°C for 30 min. After washing the oocytes in PBS-PVA three times, images were captured by using a fluorescence microscope, and the fluorescence intensity was analyzed by using ImageJ software. The average MMP of oocytes was calculated as the ratio of red fluorescence intensity (corresponding to activated mitochondria) to green fluorescence intensity (corresponding to inactive mitochondria).

### Intracellular ATP levels assay

Intracellular ATP levels were measured using an ATP assay kit (Beyotime, S0027). Briefly, 50 oocytes were collected from each group into a 1.5 ml centrifuge tube containing 45 μL of lysis buffer. The cells were lysed by ultrasound and centrifuged at 12,000 rpm for 5 min at 4°C. The supernatant was taken for subsequent determination. Then, 100 μL of ATP working solution and 20 μL of supernatant were added to a 96-well opaque plate, and the mixtures were measured by a microplate reader. The intracellular ATP levels were calculated according to the measured value and the standard curve.

### Intracellular senescence-associated β-galactosidase (SA-β-gal) activity assay

Intracellular SA-β-gal activity was measured by using a Cellular Senescence Detection Kit - SPiDER-βGal (SG03, Dojindo, Japan). Briefly, oocytes were cultured in an environment under 38.5°C and 5% CO2 for 1 h after adding 1 mL of Bafilomycin A1 working solution. Subsequently, 1 mL of SPiDER-βGal working solution was added, and the oocytes were cultured in an environment under 38.5°C and 5% CO2 for 30 min. After washing three times in PBS-PVA, the fluorescence intensity of each group of oocytes was captured by fluorescence microscope and photographed, and the fluorescence intensity was analyzed by ImageJ software.

### Sperm binding assay

Straw frozen semen was removed from liquid nitrogen and thawed. The purified sperm were obtained by density gradient centrifugation on Percoll and resuspended so that the sperm density was 1 × 10^6^/mL. Oocytes and resuspended sperm were co-incubated in IVF drops in an environment under 38.5°C and 5% CO2 for 1 h. Then, they were fixed in PBS-PVA containing 4% paraformaldehyde for 30 min. After fixation, PBS-PVA was used to wash three times, after which the samples were transferred to 10 μg/mL Hoechst 33343 to label the spermatid nuclei. Afterward, the stained sperm-oocyte complexes were mounted onto glass slides, examined and photographed by a microscope under fluorescent light. The sperm number was analyzed by ImageJ software.

### Immunofluorescence staining

Oocytes were fixed in PBS-PVA containing 4% paraformaldehyde for 30 min and permeabilized in 0.3% Triton X-100 at room temperature for 15 min. The oocytes were then blocked in PBS-PVA containing 3% BSA at room temperature for 2 hours. Next, the oocytes were incubated with a primary anti-H2AX antibody (9718S; CST, USA) at 4°C overnight. After first antibody incubation, the oocytes were washed three times in PBS-PVA, and the oocytes were incubated with goat anti-rabbit IgG (CST; 4413S for H2AX staining) at room temperature for 2 hours. Afterward, the oocytes were transferred to 10 μg/mL Hoechst 33343 at room temperature for 15 min. Fluorescence microscopy was used to determine the positivity and negativity of γ-H2AX.

### Western blot

For Western blotting, 70 oocytes were collected and lysed in SDS lysis buffer (40% ddH2O, 20% glycerol, 20% SDS, 12.5% 0.5 M Tris-HCl, 20 mM β-mercaptoethanol and trace bromophenol blue) and incubated in a 95°C metal bath for 10 min. Next, the total protein was separated by sodium dodecyl sulfate-polyacrylamide gel electrophoresis (SDS-PAGE) and transferred to a polyvinylidene fluoride membrane (Millipore, Billerica, MA, USA). Blocking buffer (WLA066a, Wanleibio, China) was used to block the transferred membrane, and the membrane was incubated overnight with first antibodies against β-actin (CST, 4970T), Bcl2 (Proteintech, 12789-1-AP), BAX (Proteintech, 50599-2-Ig) and Caspase-3 (CST, 9662S) at 4°C. After washing in TBST 3 times for 10 min each time, the membrane was incubated with goat anti-rabbit IgG (CST, 7074S) at room temperature for 1 hour. The images were analyzed with a Tanon 5200 image analyzer (Tanon, China), and ImageJ software was used for visualization and analysis.

### Statistical analysis

All the above experiments were repeated at least three times. The statistical results are expressed as the mean ± standard error of the mean (SEM). The total number of oocytes used in each experiment (n) is shown by the bar. The number of independent repetitions (*R*) is shown in the diagram annotation. Statistical analysis was performed by one-way analysis of variance (ANOVA). All statistical analyses were performed using SPSS version 22.0 (IBM, Chicago, IL, USA) software. Significant differences are expressed as (^*^P < 0.05), (^**^P < 0.01) and (^***^P < 0.001).

## Supplementary Materials

Supplementary Figure 1
