# Supplementary material for: Ferulic acid ameliorates the quality of in vitro-aged bovine oocytes by suppressing oxidative stress and apoptosis
Source: Aging (Albany NY). 2023 Nov 8;15(21):12497–512. doi: 10.18632/aging.205193 (PMC10683616; doi:10.18632/aging.205193)
Supplement: Supplementary Figure 1 [file aging-15-205193-s001.pdf]

SUPPLEMENTARY FIGURE

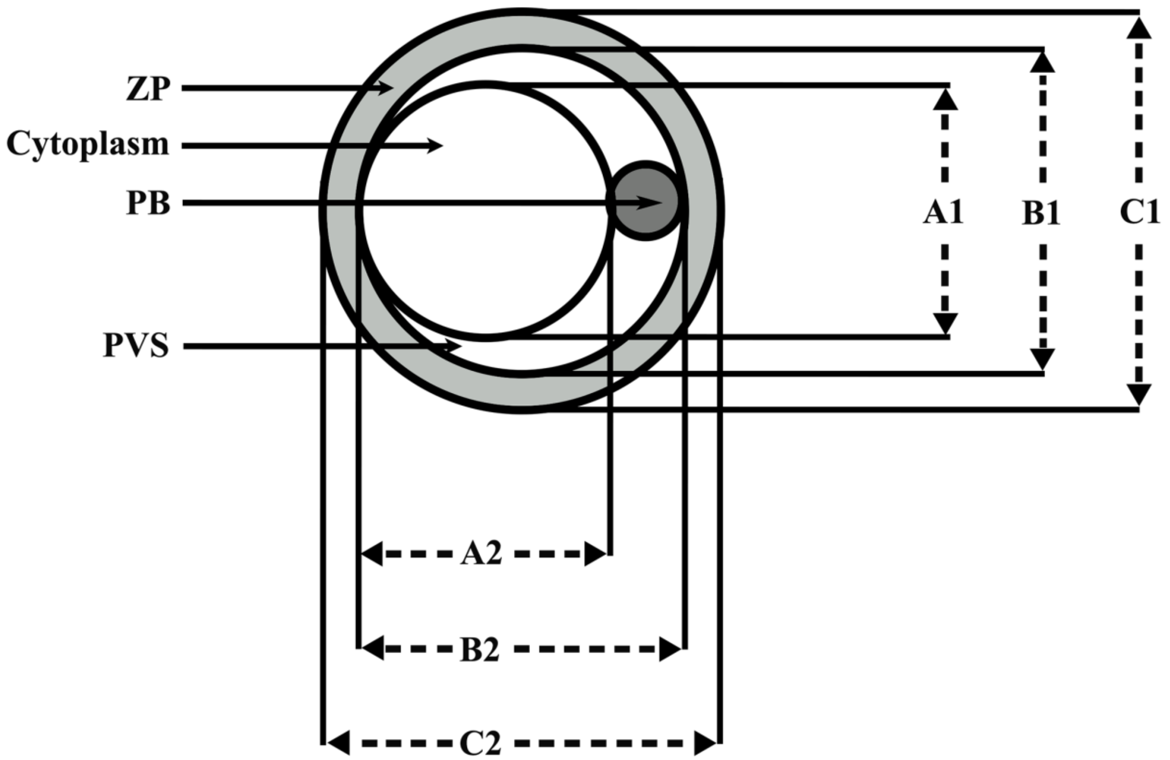

Supplementary Figure 1. Calculation method for the size of each part of the oocyte. Diameter of cytoplasm (A) = (A1 + A2)/2. Inner diameter of zona pellucida (B) = (B1 + B2)/2. Outer diameter of zona pellucida (C) = (C1 + C2)/2. Thickness of zona pellucida = (C − B)/2. Size of perivitelline space = (B − A)/2. Abbreviations: ZP: Zona pellucida; PVS: perivitelline space; PB: first polar body.
